# Deep learning-based hemodynamic prediction of carotid artery stenosis before and after surgical treatments

**DOI:** 10.3389/fphys.2022.1094743

**Published:** 2023-01-10

**Authors:** Sirui Wang, Dandan Wu, Gaoyang Li, Zhiyuan Zhang, Weizhong Xiao, Ruichen Li, Aike Qiao, Long Jin, Hao Liu

**Affiliations:** ^1^ Graduate School of Engineering, Chiba University, Chiba, Japan; ^2^ College of Life Science and Bioengineering, Beijing University of Technology, Beijing, China; ^3^ Department of Interventional Radiology, Beijing Friendship Hospital, Capital Medical University, Beijing, China

**Keywords:** carotid artery stenosis (CAS), stroke, hemodynamics, deep learning (DL), computational fluid dynamics (CFD)

## Abstract

Hemodynamic prediction of carotid artery stenosis (CAS) is of great clinical significance in the diagnosis, prevention, and treatment prognosis of ischemic strokes. While computational fluid dynamics (CFD) is recognized as a useful tool, it shows a crucial issue that the high computational costs are usually required for real-time simulations of complex blood flows. Given the powerful feature-extraction capabilities, the deep learning (DL) methodology has a high potential to implement the mapping of anatomic geometries and CFD-driven flow fields, which enables accomplishing fast and accurate hemodynamic prediction for clinical applications. Based on a brain/neck CT angiography database of 280 subjects, image based three-dimensional CFD models of CAS were constructed through blood vessel extraction, computational domain meshing and setting of the pulsatile flow boundary conditions; a series of CFD simulations were undertaken. A DL strategy was proposed and accomplished in terms of point cloud datasets and a DL network with dual sampling-analysis channels. This enables multimode mapping to construct the image-based geometries of CAS while predicting CFD-based hemodynamics based on training and testing datasets. The CFD simulation was validated with the mass flow rates at two outlets reasonably agreed with the published results. Comprehensive analysis and error evaluation revealed that the DL strategy enables uncovering the association between transient blood flow characteristics and artery cavity geometric information before and after surgical treatments of CAS. Compared with other methods, our DL-based model trained with more clinical data can reduce the computational cost by 7,200 times, while still demonstrating good accuracy (error<12.5%) and flow visualization in predicting the two hemodynamic parameters. In addition, the DL-based predictions were in good agreement with CFD simulations in terms of mean velocity in the stenotic region for both the preoperative and postoperative datasets. This study points to the capability and significance of the DL-based fast and accurate hemodynamic prediction of preoperative and postoperative CAS. For accomplishing real-time monitoring of surgical treatments, further improvements in the prediction accuracy and flexibility may be conducted by utilizing larger datasets with specific real surgical events such as stent intervention, adopting personalized boundary conditions, and optimizing the DL network.

## 1 Introduction

Stroke is a high-risk medical condition that seriously threatens human life. For the adverse consequences of stroke, brain cells and tissues can degenerate or die within a few minutes owing to insufficient oxygen and nutrient provision caused by the interruption or reduction of the blood flow from the carotid artery flow to the brain (Hachinski, 2015; Zhang et al., 2020). Stroke can be of two types based on the causality: ischemic stroke and hemorrhagic stroke, and according to clinical statistics, ischemic stroke accounts for a large proportion (about 87%) (Pan et al., 2016; Barthels and Das, 2020). The primary reason for ischemic stroke is the blockage of the common carotid artery (CCA) or internal carotid artery (ICA) induced by atherosclerosis, also known as carotid artery stenosis (CAS), which causes intracranial reduced blood supply (Alagöz et al., 2016; Brinjikji et al., 2016; Kubota et al., 2021) and usually requires revascularization surgery to prevent ischemic stroke for the patients with severe CAS. Revascularization surgeries for CAS mainly include carotid endarterectomy, carotid angioplasty, and carotid stenting (Bonati et al., 2015; Moresoli et al., 2017; Powers et al., 2019; Halliday et al., 2021). While the operating procedures of these three surgeries are different, i.e., by removing the plaque through surgery, temporarily expanding the stenotic lumen with a balloon, and placing an adaptive vascular stent after balloon dilation, respectively, the ultimate goal of the three surgeries is to enlarge the flow cavity enabling blood flowing through the stenosis to improve the insufficient blood supply problem for preventing ischemic stroke (Bandyk, 2020; Darwal et al., 2020; Doenges and Reed, 2020; Wang et al., 2021). The clinical diagnosis and postoperative prognosis of these surgeries often need to be guided by multiple hemodynamic variables such as pressure, velocity and wall shear stress (Liang et al., 2015; Wu et al., 2020), which are utilized to diagnose the severity of CAS and evaluate the surgical effect.

To accurately predict the hemodynamic characteristics for the diagnosis of cardiovascular diseases and the prognostic assessment of various revascularization surgeries, computational fluid dynamics (CFD) has now been widely used as an efficient method (Fu et al., 2010; Matsuura et al., 2018; Xu et al., 2018; Kazantsev et al., 2020; Wu et al., 2020; Han et al., 2021). CFD modeling is normally conducted in three-fold (Liu et al., 2015): 1) pre-processing to construct three-dimensional (3D) anatomic/geometric models based on medical images of CT, MRI, etc. and to discretize computational domain; 2) computation of flow fields in terms of pressures and velocities by solving the Navier-Stokes equations under certain boundary conditions (Yin et al., 2018; Driessen et al., 2019; Han et al., 2021; Hou et al., 2022; Rizzini et al., 2022); and 3) post-processing to visualize flow fields while calculating hemodynamic parameters such as wall shear stresses. Thus, the CFD-based simulations are of high computational cost due to the requirements of mighty computing resources, large-scale computing time, and highly skilled experts (Yamaguchi et al., 2016; Fu et al., 2020). Moreover, the simulation is generally performed in a patient-specific manner by using the image-based geometric model for each individual under specific boundary conditions, which needs to be conducted for all patients and is usually highly time-consuming (Fu et al., 2010; Conti et al., 2016; Bluestein, 2017; Polanczyk et al., 2018; Albadawi et al., 2021). Thus, it is a crucial issue to pay the expensive computational costs for real-time simulations of complex blood flows in association with the realistic clinical applications of CFD methods for surgical treatments such as CAS.

Given the powerful feature-extraction capabilities in multidomain regression and pattern recognition, both machine learning (ML) and deep learning (DL) methods have shown successful applications in various fields, such as physiological signal diagnosis, medical image separation, smart medical care, etc (LeCun et al., 2015; Li et al., 2019; Mittal et al., 2019; Noorbakhsh-Sabet et al., 2019; Bhandary et al., 2020; Li et al., 2021b; Wang et al., 2022). The ML and DL-based methodology is also considered as an alternative to the CFD method for blood flow analysis (Taebi, 2022) because it is of high potential to implement the mapping of anatomic geometries and CFD-driven flow fields, which enables accomplishing fast and accurate hemodynamic prediction for clinical applications. Recently, the ML/DL models have been verified capable of predicting the reduced-order simulation results in a computationally inexpensive way when merely employing some limited flow information, i.e., the velocities and pressures at the centerline or cross-section of a vessel (Itu et al., 2016; Sklet, 2018; Sarabian et al., 2021). However, from the viewpoint of clinical applications, an accurate prediction of the detailed information on 3D and transient local flows before and after surgical treatments is needed to provide sufficient clinical references for surgery-decision making, which remains poorly studied yet. With a high goal of the diagnosis of CAS disease and the effect prognosis of surgical treatments, we applied the DL methodology to the CAS disease to accomplish a fast and accurate prediction of the hemodynamic characteristics in association with carotid stenotic artery before and after the surgical operation due to the flow cavity variation. With consideration of the intense vortical flow structures induced by the complex morphology of carotid bifurcation and stenosed carotid arteries (Gallo et al., 2015; Singh et al., 2016), a flexible data format is thus employed, which is capable of accurately mapping both the carotid artery geometry and the complicated flow field.

The data format utilized in DL and ML methods is usually given in terms of pixels or voxels to deal with the irregular shape and connectivity information, which has resolution limitations in accurately representing the complex arterial geometry and hence reasonably predicting the CAS hemodynamics *via* CFD simulations (Guo et al., 2016; Liang et al., 2020). While there still exists the accuracy issue in the boundary representation (BRep) with smoothened boundaries, the point cloud dataset has the advantage of being easily generated through converting and transforming from a 3D scanned dataset by means of CAD conversion software (e.g., Solidworks, United States) (Spatial Team, 2019). The point-cloud data format enables the characterization of both complex geometry of the vessel model and the complicated flow fields with high resolution; and the high-density point cloud capable of conducting potential feature-extraction can be achieved with a small size dataset (Qi et al., 2017; Kresslein et al., 2018; Li et al., 2021b; 2021a). Furthermore, a novel DL network can be employed using dual input-sampling channels, which enables the high-performance analysis and establishment of the correlation between arterial geometries and velocity and pressure fields through abstracting and incorporating global and local characteristics of the point cloud dataset (Li et al., 2021b; 2021a).

In this study, a total of four point-cloud datasets were established and utilized to validate the CFD simulations and perform the hemodynamic prediction of the CAS models before and after surgical treatments in terms of the flow cavity variation. To match the CFD-based point cloud datasets, we employed a DL network with dual input-sampling channels. After the DL training, the optimal weight configurations were stored for the DL-based hemodynamics prediction of the CAS in the testing process. Compared with other methods, the evaluation of prediction performance and the DL analyses indicated that the DL strategy proposed here enables uncovering the association between transient blood flow characteristics, including velocity and pressure fields and artery cavity geometric information before and after surgical treatments of CAS. A remarkable reduction of 7,200 times is achieved in the computational cost, and the DL-based predictions are well consistent with the CFD simulations in terms of mean velocity in the stenotic region for both the preoperative and postoperative datasets. Our study thus points to the potential and feasibility of the CFD-driven, DL-based methodology in predicting the 3D and transient hemodynamics associated with CAS before and after treatments, which may provide an effective and useful tool for the diagnosis of ischemic stroke and prognosis of surgical treatments.

## 2 Methods

### 2.1 Ethics approvals

This prospective study was conducted in compliance with the principles of the Declaration of Helsinki and met the requirements of medical ethics. The Ethical Review Committees of Beijing Friendship Hospital approved this research. All measurements and collection of the data were carried out under relevant regulations and guidelines. We obtained signed informed consent forms.

### 2.2 Clinical data collection

All clinical data used in this study were taken from Beijing Friendship Hospital. The raw CTA data of the carotid arteries for 298 subjects who visited Beijing Friendship Hospital in 2021 and 2022 to examine the cerebral and carotid arteries were collected and collated by professional clinicians with 128^−ΔΔCT^ (Brilliance iCT, Philips Health care, Netherlands). In addition, technicians reconstructed 3D anatomic models by importing the CT images into MIMICS 20.0 (MIMICS, Leuven, Belgium) for arterial segmenting and repairing. Eventually, 280 3D geometric models with no stenosis of carotid bifurcate arteries were built up, and among them 18 heterogeneous cases were excluded due to incomplete information.

### 2.3 Preoperative and postoperative CAS models

It was difficult to perform accurate and efficient DL analyses on the hemodynamic characteristics in association with geometric features of carotid artery stenosis (CAS) by using the mere 280 realistic carotid artery models. Moreover, most patients were found not suffering from surgery treatment for CAS. On the other hand, it has been recognized that the key parameters significantly impacting the CAS hemodynamics consist of the diameter of common carotid artery (CCA), the diameter of internal carotid artery (ICA), the diameter of external carotid artery (ECA), the bifurcation angle between ICA and CCA, the stenosis location, the number of stenoses, the stenosis severity, and the stenosis length (Smith et al., 1996; Goubergrits et al., 2002; Thomas et al., 2005; Spanos et al., 2017). Therefore, with the clinicians’ agreement and on the basis of the 280 CA models, we reconstructed more models artificially through adjusting these seven parameters as summarized in Table 1, and substantially built up 1,000 geometric models. It is worth noting that for the 1,000 geometric models, the CAS models were then constructed by randomly changing the stenosis-related parameters within a given range (Table 1) using the modeling software SolidWorks 14.5 (Solidworks, United States), substantially resulting in a dataset of 1000 CAS models.

**TABLE 1:** Geometric parameters of carotid arteries and stenoses.

Parameter	Description	Range
Diameter of CCA	Increased or decreased the diameters of the original artery uniformly	6.7–9.0 mm
Diameter of ICA	Increased or decreased the diameters of the original branch artery (for brain) uniformly	4.6–6.3 mm
Diameter of external carotid artery (ECA)	Increased or decreased the diameters of the branch artery (for face and ears) uniformly	3.8–5.2 mm
Bifurcation angle between ICA and CCA	The angle formed by the two branches in the first 10 mm of their course was measured	20–120°
Stenosis location	Random positions on ICA and CCA	
Number of stenosis	ICA and CCA	1–2
Stenosis severity	Severity of stenosis	0%–80%
Stenosis length	The length of the stenosis on the ICA or CCA	5–20 mm

### 2.4 CFD simulation of CAS before and after surgical treatments

**FIGURE 1:**
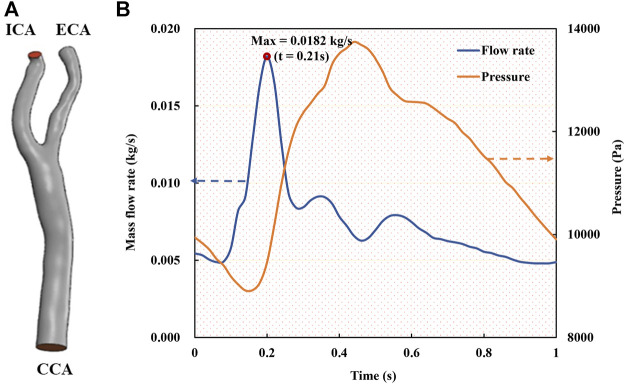
Geometric model and boundary conditions of the carotid bifurcated artery. **(A)**. Geometric model of carotid bifurcate artery with branches CCA, ICA and ECA; **(B)**. Unsteady boundary conditions comprising a mass flow rate profile at CCA inlet and a pressure waveform at ICA and ECA outlets (Gharahi et al., 2016; Lopes et al., 2019).

After geometric model augmentation, we carried out a series of CFD simulations to resolve the flow fields and make the hemodynamic prediction for the 1000 CAS models (Figure 1A). The blood flow was treated as an incompressible, laminar, and Newtonian viscous fluid with the density of 1,060 kg/m^3^ and the viscosity of 0.0035 Pa·s (Gharahi et al., 2016; Lopes et al., 2019). All arterial walls were treated as rigid boundaries and the nonslip condition was imposed. The commercial software ANSYS-Meshing was utilized for discretizing the computational domains in terms of the tetrahedral mesh with a minimum size of 0.0455 mm. The mesh independency convergence analysis was conducted in terms of the minimum mesh spacing adjacent to the walls and the mesh number, and it was verified that the results (see details in the Results section) were well consistent with the previous studies (Lopes et al., 2019).

At the inlet of the CCA models, a pulsatile mass flow rate profile (Figure 1B) with the waveform taken from the previous studies (Gharahi et al., 2016; Lopes et al., 2019) was defined, which was spatially uniform while pulsating with time. A transient pressure waveform was simultaneously imposed at the outlets of the ECA and ICA. Given the dimension of the CCA part with a cross section area of 45.23 mm^2^ and a diameter of 7.318 mm, and the average flow velocity in a cardiac cycle (Figure 1A), the Reynolds number was calculated to be approximately 346. It is worth noting that a peak Reynolds number based on the peak mass flow rate (0.182 kg/s) among all our models was approximately 2,200. The numerical simulations were performed with ANSYS-CFX 16.0 (ANSYS, Canonsburg, United States) by solving the unsteady Navier-Stokes equations and the continuity equation. The time step was set as 0.01 s, and the maximum iteration number was set to 200 for each time step, which was confirmed capable of ensuring a numerical convergence with the residuals less than 10^–4^. In addition, all the simulations were performed up to four cardiac cycles, when the flow field was confirmed to reach a stable and converged state. The results of the fourth cycle were used for further hemodynamic analysis.

### 2.5 Creation of DL datasets

This study is attributed to developing a DL strategy to implement the mapping of anatomic geometries and CFD-driven flow fields to achieve the hemodynamic prediction of 3D carotid artery stenosis (CAS) before and after surgical treatments. Thus, both creations of the DL datasets and the construction of a suitable DL network play crucial roles. The point cloud data were employed herein to characterize the 3D CAS models (mesh nodes). The point clouds of two types were extracted from the CFD-based results, representing the geometric features of the CAS cavity and the hemodynamic characteristics, respectively. A suitable DL network with dual input and sampling channels was then developed and employed for the DL analysis.

Because the flow field data comprising velocities and pressures at each mesh obtained through ANSYS software can be directly converted into a high-density point cloud data, we extracted all the CFD results at the instant of 0.21 s, i.e., the systole peak of the fourth cycle. We then established two types of point clouds, namely, the cavity point cloud {N_1_*P_1_} extracted from the innermost layer of the carotid artery wall (i.e., geometric information of flow cavity) and the fluid point cloud {N_2_*P_2_} extracted from the inside of the CA model. Here, N_1_ denotes the total number of grids in the lumen shell, P_1_ denotes the coordinate information of the carotid lumen, N_2_ denotes the total number of grids of the internal fluid, and P_2_ denotes the comprehensive properties of the internal fluid, including the information of spatial coordinates and flow fields of velocity and pressure.

In general, any variations in the 3D CAS models would alter their wall surface meshes and hence the mesh distributions in the computational domain, substantially resulting in the change of the spatial distribution of point cloud. The point cloud data thus consists of both the geometric information of the spatial coordinates (x, y, z) and the corresponding CFD-based flow field information of velocities and pressures, which can be collected and stored simultaneously at each discrete point of the point cloud data (Raissi et al., 2020; Li et al., 2021b; 2021a).

We built up four datasets of the CAS models in terms of either velocity field data or pressure field data, with two preoperative datasets for before surgical treatment and two postoperative datasets for after surgical treatment with the cavity geometry changed, which are summarized in Table 2. All samples in the four datasets contain both fluid point clouds and cavity point clouds. After the establishment of the four datasets, we randomly divided each dataset into a training set and a testing set with a ratio of 9:1 for DL analysis. Thus, each training set includes 900-point cloud sets from CFD simulation results, and each testing set includes 100-point cloud cases. These four datasets were used for training and testing in four independent DL networks.

**TABLE 2:** Four DL datasets.

Stage	Hemodynamic	Training number	Testing number
Preoperative	Velocity	900	100
	Pressure	900	100
Postoperative	Velocity	900	100
	Pressure	900	100

### 2.6 DL network

**FIGURE 2:**
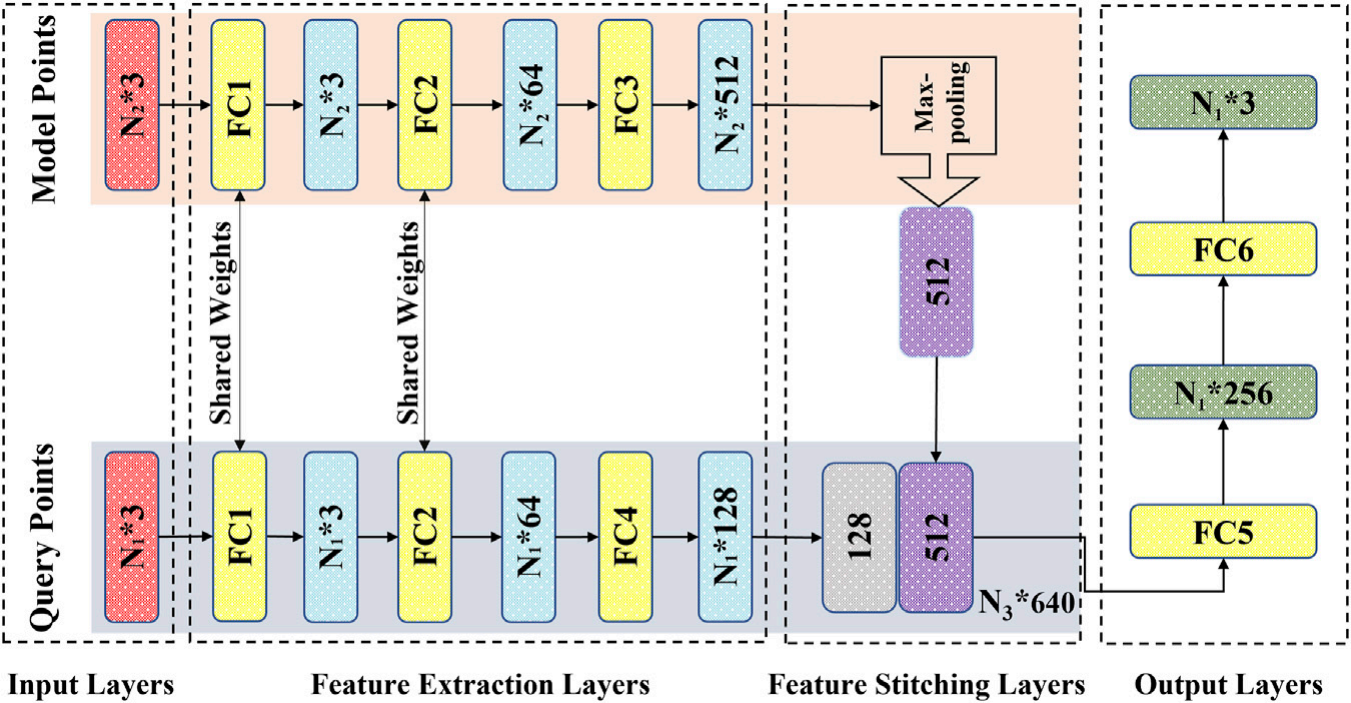
Structure of the proposed network.

According to the characteristics of the established point cloud datasets, we employed a matching dual-input-sampling channel DL network. As shown in Figure 2, the network has two inputs and sampling channels that receive and process the overall outer cavity and inner fluid point clouds of the carotid artery model, respectively. For the sampling module, to enhance the correlation between the point clouds of the two channels while improving the network prediction performance, the first two feed-forward fully connected layers, i.e., FC1 and FC2 (Figure 2) are utilized to share the weights, i.e., the same preliminary feature extraction method. After the step of FC2, the two types of point clouds enter their respective independent feed-forward fully connected layers, i.e., FC3 and FC4 (Figure 2). They are used to extract the overall features from the outer cavity point cloud and to characterize the flow field information from the inner fluid point cloud. After being processed by the sampling module, the characteristics of both outer cavity geometry and the inner fluid flow are extracted as 512-dimensional {N_1_ * 512} and 128-dimensional {N_2_ * 128}-dimensional vectors, respectively, which are first encoded in the feature stitching module as a {N_3_ * (512 + 128) = 640} dimensional vector. Then, the dimensional vector {N_3_* (512 + 128) = 640} containing the two characteristics in the output module (FC5 and FC6) is decoded into {N_2_, P_2_}, i.e., the flow field information of the internal fluid, which functions as a convolutional neural network decoding operation. By employing the network with the two matched point clouds to bridge the fluid’s overall cavity and spatial coordinates, the flow field data of velocity and pressure at each point, can be substantially determined.

With the utilization of a dual-channel rather than a single-channel and sharing weights in the fully connected layers instead of nonshared weights, Li (Li et al., 2021b) investigated the prediction performance through testing with control variables in previous work. Here, we focused on the feature extraction and processing point clouds over the network. We added a max pooling part in the sampling module as a symmetric function (Figure 2) to resolve the disorder issue of the input point clouds (Qi et al., 2017; Ghahremani et al., 2020). In addition, the mean absolute error (MSE) was chosen as the loss function**;** the Adam optimizer was utilized as a learning rate = 0.001, ε = 0.001, ρ1 = 0.9, ρ2 = 0.999, and δ = 1E−8 (Kingma and Ba, 2014; Li et al., 2019).

### 2.7 Network training and testing

Four DL datasets were trained separately using independent networks in the environment of TensorFlow (v2.0.0rc, Python3.7) on an Nvidia GeForce GTX 1660 Ti GPU with a batch size of 1 and epoch of 1,000. In the training phase, we stored the optimal weight configuration by optimizing the loss function to the minimum value, which resulted in four trained networks for the DL prediction at the testing stage. For the testing phase, the hemodynamic results of fluid points in P_2_ were predicted by only importing the spatial coordinated information of the cavity point cloud in P_1_ and the spatial coordinate information of the fluid point cloud in P_2_ using the stored optimal configuration.

### 2.8 Evaluation of prediction performance

To quantitatively evaluate the difference between the DL-predicted results and the CFD simulation results, we drew on previous studies to employ the mean radial error (MRE) and the normalized mean absolute error (NAME) to determine the error at each mesh point (Liang et al., 2020; Li et al., 2021b; 2021a). MRE can characterize the error of the DL prediction value relative to the actual value at all query points of the model. The NMAE can characterize the error of the DL-based result relative to the actual value of the overall flow field (CFD result). The definitions of MRE and NAME are given in Eqs 1, 2:

$$MRE\left(y,\hat{y}\right)=\frac{1}{N_{2}}\frac{\sum_{i=1}^{N_{2}}\sqrt{{\left(y_{i}-{\hat{y}}_{i}\right)}^{2}}}{\sqrt{{y_{i}}^{2}}}\times 100\%,$$ (1)

$$NMAE\left(y,\hat{y}\right)=\frac{1}{N_{2}}\frac{\sum_{i=1}^{N_{2}}\left|y_{i}-{\hat{y}}_{i}\right|}{\mathrm{Max}\left|y\right|-\mathrm{Min}\left|y\right|}\times 100\%,$$ (2)

where $y_{i}$ and ${\hat{y}}_{i}$ denote the *ith* inner fluid point values of pressure or velocity obtained by DL-predicted values and CFD-simulated results, respectively. *I* is the point spatial sequence. N_2_ is the total number of fluid point clouds. Max|y| and Min|y| represent the maximum and minimum magnitudes of the corresponding hemodynamic parameters among all points in the selected area, respectively.

## 3 Results

**FIGURE 3:**
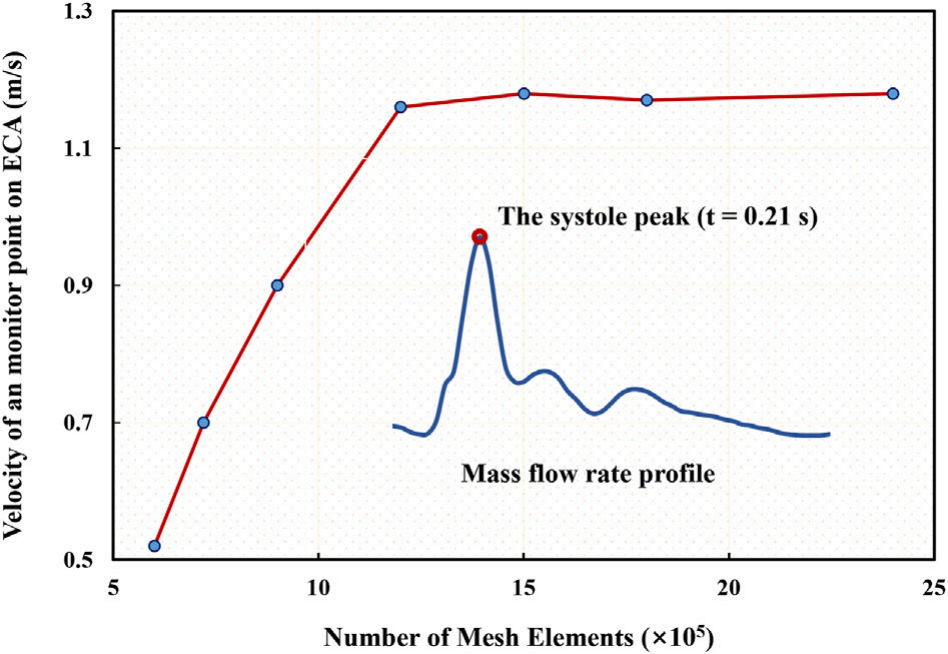
Mesh convergence analysis in terms of mesh independency associated with a specific velocity at the systole peak of the fourth cycle.

First, the mesh convergence analysis was conducted by investigating the mesh independency of the CFD simulation in terms of minimum mesh spacing adjacent to the wall and mesh number. For the sake of simplicity, a stenotic carotid artery model, as shown in Figure 1A, was used for the mesh convergence analysis. It was verified that a minimum mesh spacing/size of 0.0455 mm of the tetrahedral meshes at the wall surface was good enough to capture the hemodynamic characteristics of the flow field accurately; and the mesh number exceeding 1.2 million could achieve a marginal difference in association with the velocity magnitude at the systole peak (Figure 3), less than 3% with increasing the mesh number. With consideration of the balance between numerical accuracy and computational cost for the CFD simulation, we thus selected the number of mesh nodes (point cloud) ranging from 0.18 to 0.25 million, identical to a mesh number exceeding 1.2 million in total across different cases, which were verified capable of accurately and effectively representing the geometric features and flow field details of the CAS models.

**FIGURE 4:**
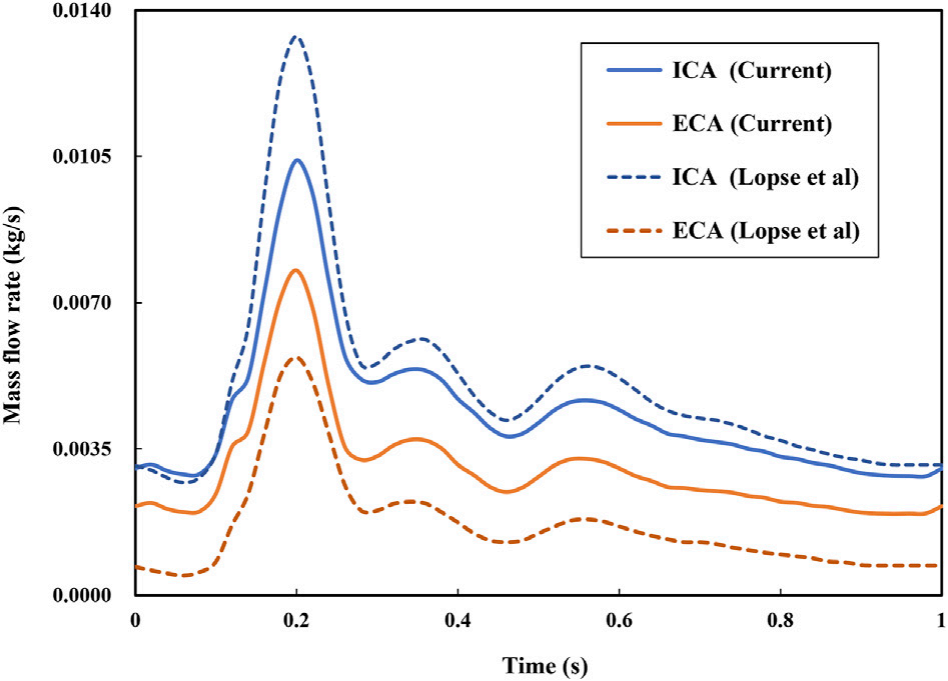
Comparison of simulated mass flow rates at two outlets with published data (Lopes et al., 2019).

Because the DL analyses in terms of accuracy and validity are highly dependent upon the data quality, particularly in the present case of the unsteady flow field, which could exert a significant impact on the point cloud data converted by CFD simulation results. Therefore, we validated the time accuracy of the CFD simulation through a comparison of the mass flow rate at the ICA and ECA outlets in Figure 4. The current CFD-based results are in reasonable agreement with reliable published data (Lopes et al., 2019) in terms of the time-varying mass flow rates at the two outlets of the carotid model even though some noticeable differences exist in the amplitudes mainly due to the discrepancy in the two models.

**FIGURE 5:**
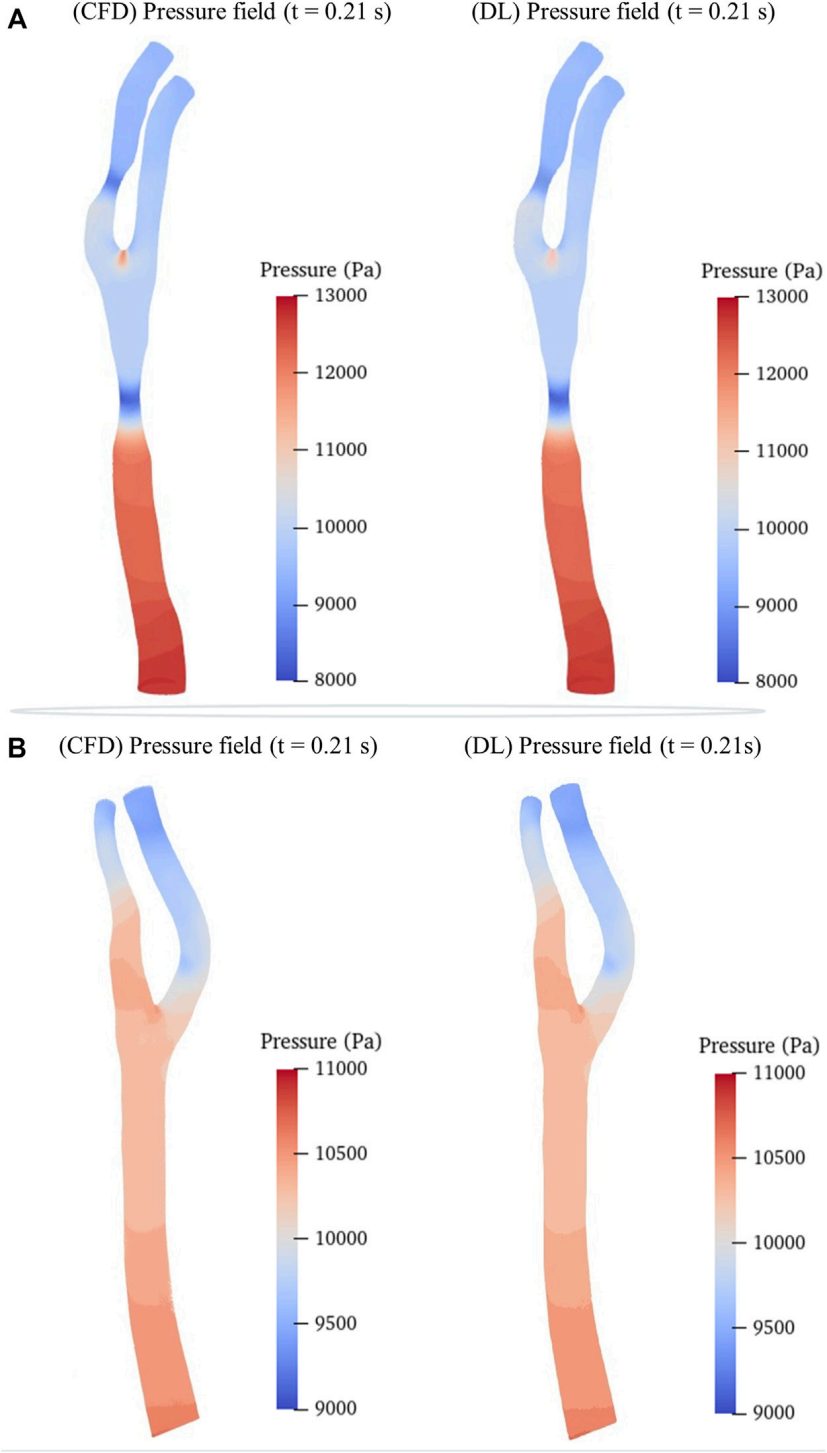
Comparison of pressure fields between CFD simulation and DL prediction. **(A)**. Carotid stenotic artery model; **(B)**. Carotid artery model without stenosis (cavity changed model).

**FIGURE 6:**
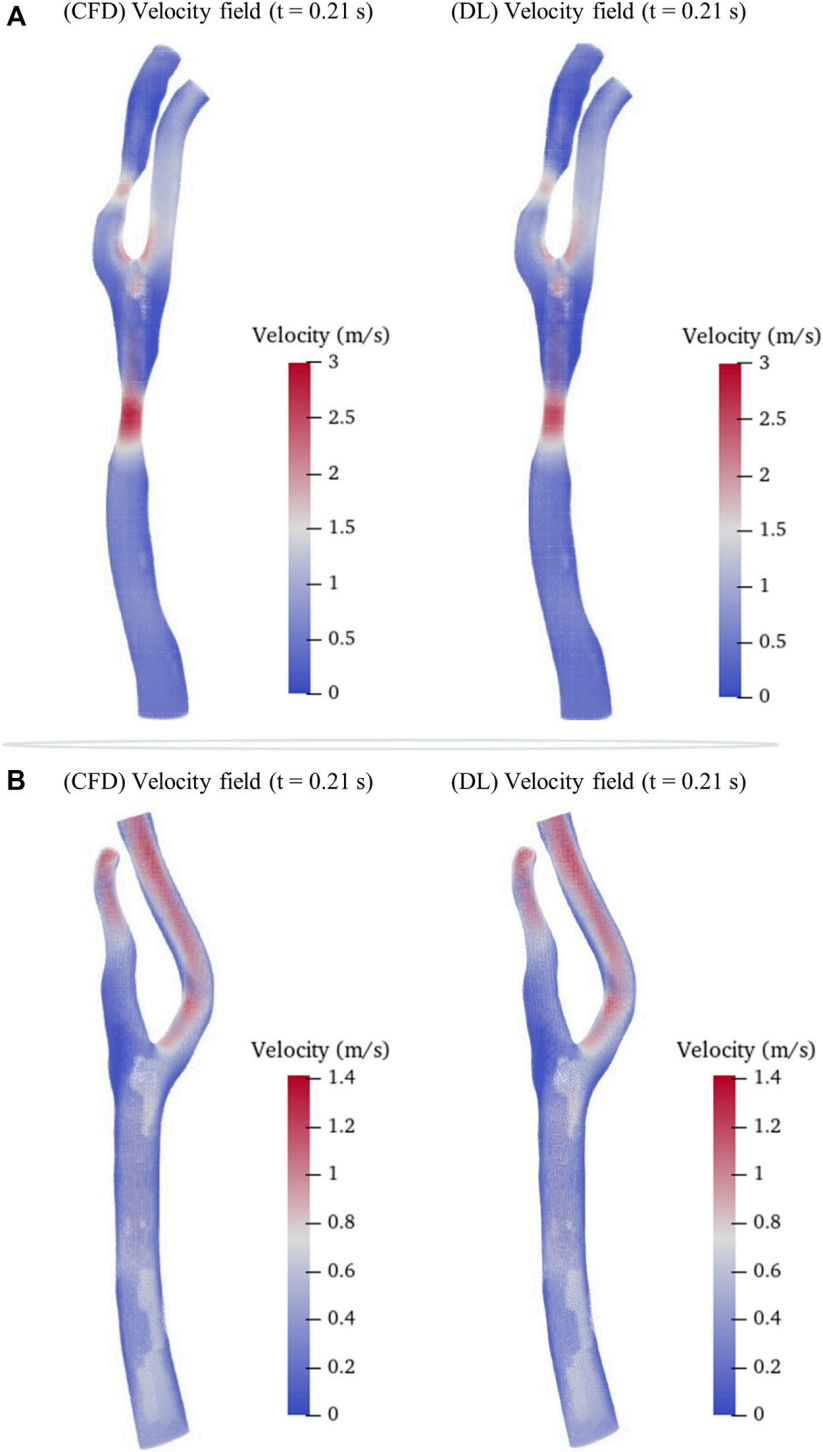
Comparison of velocity fields between CFD results and DL prediction. **(A)**. Carotid stenotic artery model; **(B)**. Carotid artery model without stenosis (cavity changed model).

We then randomly selected a preoperative model and a postoperative (cavity changed) model from the testing sets as samples to intuitively illustrate the predicted hemodynamic results in terms of pressure and velocity distributions of the maximum inflow rate (*t* = 0.21 s in Figure 1B) as illustrated in Figures 5, 6. It is observed that both the pressure fields (Figure 5) and velocity fields (Figure 6) associated with the CAS model and the normal carotid artery model (i.e., the cavity changed model) display excellent consistency between the CFD-based and DL-predicted results.

In addition, we summarized the error function results of the velocity and pressure fields in Table 3 in terms of the mean radial error (MRE) (Eq. 1) and the normalized mean absolute error (NAME) (Eq. 2) to investigate the error at each mesh point of the testing set models. Except for the overall error, we also segmented the narrow stenotic portion of the CAS model and calculated the corresponding errors. The error function results indicate that our DL method can achieve reasonable and effective hemodynamic prediction with the maximum error of less than 12.5% throughout the flow field inside the CAS model. The prediction errors for the pressure field are noticeably lower than those of the velocity field, which may be due to the DL-based prediction of the three velocity components at each point can significantly increase the computational cost associated with the corresponding network, substantially leading to high errors. Besides, the errors of the stenotic models are noticeably larger than the normal carotid artery model (the cavity changed model), probably because of the complex transient flow structures in the vicinity of the stenosis, which may lower the DL-based prediction accuracy. With respect to the computational cost between CFD simulation and DL-based prediction, it is obvious that the DL method is superior, which enables the prediction to be accomplished within merely 1 s. The CFD simulation that comprises the pre-processing of the geometric CAS modeling, the numerical simulation for four beat cycles, and the post-processing of the computed results, however, being run at Intel Core I5-9400 2.9 GHz × 4 CPU, takes approximately 2 h on the server, indicates that the computational cost of the CFD simulation can be reduced by approximately 7,200 times.

**TABLE 3:** Error functions of pressure and velocity fields.

Types	Locations	Hemodynamic parameters	NMAE	MRE
Preoperative	Whole Model	Pressure	3.34 ± 1.31	6.47 ± 1.42
		Velocity	4.53 ± 1.45	8.03 ± 1.57
	Stenosis	Pressure	6.31 ± 2.72	10.42 ± 3.69
		Velocity	7.16 ± 1.50	11.48 ± 3.86
	Bifurcation	Pressure	5.34 ± 1.69	10.63 ± 2.64
		Velocity	6.88 ± 2.25	12.35 ± 3.61
Postoperative	Whole model	Pressure	1.77 ± 1.12	3.81 ± 1.47
		Velocity	2.83 ± 1.33	4.29 ± 1.67
	Bifurcation	Pressure	3.91 ± 2.39	7.87 ± 2.05
		Velocity	5.47 ± 1.74	9.73 ± 2.60

**FIGURE 7:**
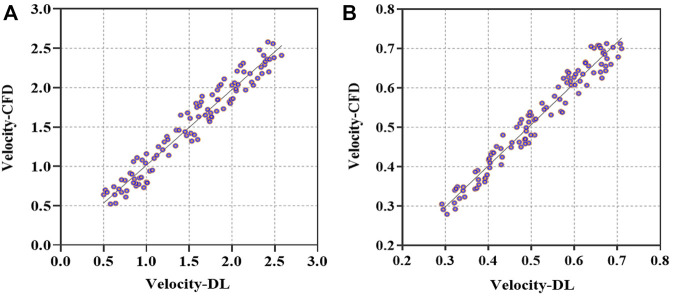
Comparison of averaged velocities in the vicinity of stenosis between DL- and CFD-based results. **(A)**. Scatterplot of averaged velocities for preoperative models in terms of Velocity-CFD and Velocity-DL. **(B)**. Scatterplot of averaged velocities for postoperative models in terms of Velocity-CFD and Velocity-DL.

In addition, we carried out a consistent analysis of the DL- and CFD-based results to examine the prediction performance and capability of clinical application. We calculated the averaged flow velocities, i.e., the mean values of velocities at all points of the narrowest cross-sections of the stenoses on ICA and CCA, based on the preoperative and postoperative testing set models. Correlations between DL- and CFD-based average velocities are compared in terms of Velocity-CFD and Velocity-DL, as depicted in Figure 7 of the scatter plot of the preoperative models with *r* = 0.9471, *p* < 0.001 (Figure 7A), and the scatter plot of the postoperative models with *r* = 0.9584, *p* < 0.001 (Figure 7B), respectively. Obviously, good consistency is observed between the DL-based predictions and the CFD-based simulations equally in the preoperative and postoperative datasets.

## 4 Discussion

In this study, we proposed a DL strategy for the first time to predict the 3D and unsteady hemodynamics of stenotic carotid arteries before and after surgical treatments (i.e., cavity change). Error analysis results show that the DL strategy can achieve high-accuracy hemodynamic prediction (ERR<12.5%) while reducing computational cost by 7,200 times, which demonstrates the clinical potential and practical capability of the DL strategy in predicting complex hemodynamics for stenotic arteries while reducing the computational cost and simplifying the operation process.

As summarized in Table 4, the previous studies on predicting flow fields or hemodynamic parameters based on ML or DL methods are limited to either 2D and reduced models or simplified 3D models but with no applications to the complex CAS. Itu et al. reported an ML-based model to predict the FFR parameter (Itu et al., 2016) but with a reduced-order model, which is highly targeted but limited in its application scope. Guo et al. presented a deconvolutional network (CNN)-based model for the prediction of 2D and/or 3D flow fields (Guo et al., 2016) by developing a dimensionality-reduction model, however, which can cause the information loss of flow fields because the data normalization process introduces considerable noises, needing larger datasets and hence much more computational cost. By generating a large number of idealized blood vessel models based on a small size of clinical datasets and employing a convolutional neural network, Su et al. achieved the 2D real-time wall shell stress (WSS) prediction (Su et al., 2020), but their model did not take account for the realistic spatial geometric information. Recently, Liang et al. built up 3D idealized thoracic aorta models but used merely 80,100 nodes for model segmentation and normalization of the human thoracic aorta (Liang et al., 2020), which was combined with a DL method. Even though a high-resolution prediction of 3D hemodynamics was achieved, the small-scale dataset of subjects and the utilization of a fixed mesh set for different geometric models largely constrained the flexibility and accuracy of the simulations.

**TABLE 4:** Comparison of ML- and DL-based methods on hemodynamic prediction.

Method	Predicting objective	Subject number	Data size	Data format	Performance
Current DL-based strategy	3D CAS unsteady hemodynamics	298	1000	High resolution point cloud	MRE <12.5%, NAME <7.5%
ML approach (Itu et al.)	Fractional flow reserve (FFR) value	87	12000	Geometric parameter	Accuracy = 99.7%
Deconvolution Network (Guo et al.)	2D steady flow	None	400000	Low resolution pixels	MRE <3%
CNNs model (Su et al.)	2D unsteady WSS distribution	small	2000	Low resolution pixels	MAE <2.5%
DNNs model (Liang et al.)	3D thoracic aorta hemodynamics	25	729	Low resolution meshes	NMAE<6.5%

Compared with previous ML- and DL-based studies, this study first manifested in larger clinical datasets, which could demonstrate better generality in terms of capabilities in clinical application. And besides, we used two formats of point cloud datasets that can flexibly characterize the stenosis/cavity geometry and carotid flow fields while employing a double input-sampling network structure for feature extraction and 3D hemodynamic prediction. The mesh-independent test result demonstrated that it is sufficient to accurately characterize the geometry of stenotic carotid artery models at a suitable resolution. The variability of point clouds regarding quantity and spatial coordinates is conducive to accurately characterizing different complex models that vary from a preoperative artery to a postoperative artery, which previous DL studies cannot handle. For instance, a stenotic model contained approximately 40,000 cavity points and 220,000 fluid points. And to match the point cloud’s characteristics, the employed DL network could extract the geometric information from the cavity point cloud while obtaining the hemodynamic information from the fluid point cloud. Specifically, the network was separately constrained by the global geometric features of the overall blood cavity while guided by the local hemodynamic information. Thus, the combination of the point cloud and the DL network could effectively introduce spatial relationships by stitching the two modules and then realizing point-by-point hemodynamic prediction of a carotid artery.

For the ERR results, the stenotic model was higher than the cavity changed model (Table 3), which may be due to the difference in the flow field and complexity of the narrow location. In our CAS models, the number of fluid points in the lesion area of the artery accounted for approximately 10% of the entire model, which means that the ERRs of the entire model was mainly determined by the stenosis part. In addition, flow field changes in the stenosis due to a narrow lumen, as well as secondary flow near the bifurcation site, lead to large changes in the flow field at the stenosis site. On the other hand, compared with the healthy model, the lesion model (CAS) has a larger range regardless of the velocity field and the pressure field, resulting in the ERRs in the stenotic part being more sensitive to flow field changes. Taking the above factors into consideration, the ERRs of diseased arteries, i.e., the CAS models, were higher than those of healthy arteries (the whole model), and the ERRs of the lesioned and bifurcated areas of the models were the highest.

The limitations of this study mainly lie in the insufficient number of clinical patients and the related pathological information. These limitations are reflected in the following: First, 720 augmented models were constructed through morphological modification of the carotid artery models based on the original dataset of the 280 patients but without apparent CAS features, which was conducted by adjusting the seven primary parameters of the diameters of CCA, ICA, and ECA, the bifurcation angle between ICA and CCA, the stenosis location, the number of stenoses, the stenosis severity, and the stenosis length base on. Thus, it is necessary to enlarge the original dataset by recruiting more patients with recognizable CAS diseases to enhance the efficiency, stability, and accuracy of the DL training analysis. Second, the lesion generated for constructing the stenotic artery is idealistic and thus ignores the diversity in stenoses such as asymmetric type, multiple contiguous types, etc. Therefore, a comprehensive analytical study on simultaneously validating the applicability and ability of our DL strategy for patients with different types of stenosis will be explored in our future studies by expanding the sample size of real clinical data. Third, instead of imposing personalized boundary conditions on each artery, we employed a generic boundary condition for the CFD simulations and then selected the CFD results for the DL dataset generation only at one time instant. Moreover, owing to solely focusing on the cavity change while ignoring the influences of specific surgical treatments, we neither utilized the arterial models treated by carotid endarterectomy nor a balloon or a vascular stent for expanding the narrowed artery. Therefore, like the postoperative scars, the thickness of an actual vascular stent, and the interaction between stents and blood vessels that we did not include in our study (Xu et al., 2018), which in turn may impact the reasonably of hemodynamic results and lead to potential errors. Finally, our study only chose the artery portion near the carotid bifurcation as the object of interest. It did not account for the cerebral artery and facial artery parts downstream of the ICA and the ECA, respectively, as well as the cardiovascular artery upstream of the CCA, which will be evaluated in our future study.

In summary, this study aimed to employ a flexible data format to represent high-resolution geometric stenotic arteries while proposing a suitable DL network and substantially achieved an accurate prediction of hemodynamic results of carotid stenotic arteries before and after surgical treatments. Therefore, with the high goal of applying our DL strategy to real-time clinical revascularization surgery guidance, improvement of our strategy prediction performance and applicability through optimizing our DL methods with larger datasets will be our next research target.

## 5 Conclusion

In this study, we proposed a simulation-based framework to achieve DL-based hemodynamic prediction of normal and diseased carotid arteries. Through establishing high-quality point cloud datasets combined with an advanced DL network, the DL-based methodology is verified capable of achieving high accurate DL predictions, which are well consistent with computational fluid dynamic (CFD) simulations while dramatically reducing computational costs. This points to the capability and feasibility of the DL-based strategy for fast and accurately predicting the hemodynamics of carotid artery stenosis (CAS) before and after surgical treatments.

## Data Availability

The original contributions presented in the study are included in the article/Supplementary Material, further inquiries can be directed to the corresponding author.
